# Dentistry as a Fine Art. No. 5

**Published:** 1867-10

**Authors:** Norman W. Kingsley

**Affiliations:** Professor of Dental Art and Mechanism, in the New York College of Dentistry.


(To be Continued.)

---

### ARTICLE IV.

### *Dentistry as a Fine Art. No.* 5.

By Norman W. Kingsley, Professor of Dental Art and Mechanism, in the New York College of Dentistry.

Our last two articles, as the reader will remember, were devoted to a consideration of the forms of teeth and their arrangement in the arch, with sole reference to their beauty and natural appearance.

Those considerations, important in themselves, are relatively of far less consequence in an artistic sense, than the subject of the present paper.

The peculiar form and position of each tooth is of minor importance compared with the contour of the whole system in its relation to the external features.

The form of each tooth may be all that art requires; the color and arrangement, of the most deceptive character; the mechanical execution, superior; and yet if the original expression of the features is not restored, or one equally pleasing substituted, the whole contrivance as a work of high art is a failure.

The highest attainment of mechanical skill, is not incompatible with the requirements of art.

All that good taste suggests, can be obtained without the violation of mechanical laws.

A thorough mastery of the mechanics, is the best foundation for the art. The mechanical and the artistic are so intimately related that it is impossible to separate all the essential conditions of art from their association with mechanical skill.

But as mechanical excellence ranks below artistic, it is not uncommon to find the requirements of the former only, fulfilled. This article therefore, like those preceeding, pre-supposes that in making an artificial denture, all that mechanical skill can perform, has been, or will be accomplished.

In the sculptor's art, every excellence desired in the finished marble, must be wrought out in the plastic clay; every thought which form or feature is required to express must provided for in this model.

In our art all this, is also done in the earlier stages of the process. The final appearance must be determined in the easily wrought model at the time that, what is technically called "the bite" is taken.

It matters not of what material the plate, or base of support be made; gold, platina or gutta percha, the same process of ascertaining the length, breadth and fulness must be prosecuted. This is the most trying and critical point in the artistic construction of the piece. On this model we may well devote anxious thought and study. Here we concentrate all our resources; display our knowledge, taste and judgement and proportionate with this exercise will be our success.

The moulding of faces into an ideal form is not alone the work of the sculptor, neither is the only material inanimate clay. The living subject in the hands of the dentist may often be changed by his skillful art from repulsive deformity into beauty and loveliness. Not only may the organs of mastication be replaced, together with the charm which, (when well formed) their presence always gives; but haggard and sunken cheeks may be made young again, lips contracted and expressionless from want of support may be arched and made full, a chin whose character has been destroyed by an alteration in the associated features will have its character restored the mouth will again receive the power of becoming eloquent and even the nose will share in this pleasing transformation.

Thus nose, cheeks, mouth and chin, may be modeled over, and again made to approach the divine.

It is not the insertion of teeth, however beautifully formed, that will work this revolution. The most perfect substitutes ever conceived may be so ill-adapted as to increase the deformity and cause an expression at variance with the character and a lasting libel upon it.

The alveolar processes which supported the muscles of the face and thus contributed to its expression ; now wasted and gone, must have a substitute and no unskillful hand is required to model up this form.

In re-modeling this living face all the resources of ideal art should be at our command. The esthetic sense should be cultivated by a familiarity with the best productions of art in all ages, together with a knowledge of the feelings and motives which prompted their production.

It was a favorite effort with the Greek sculptors to represent man deified. In the form and expression of their gods they rejected as far as possible the grosser characters of the human countenance. Their deities were built upon the human mould, for the finite mind can have no higher conception of a form possessing moral and intellectual attributes, than that, which the Great Creator has given to his noblest work, man.

To idealize this head therefore, and give it the attributes of the divine, unclouded by human infirmities constituted the highest exercise of their talents.

By what process of reasoning this was accomplished will be most interesting to note.

It has been maintained by some that this can only be the result of long acquaintance with the human face, until the artist has acquired that good taste and judgement which enables him to select from a variety of individuals and combine in one representation, a collection of beauties. This would certainly produce an ideal head of great physical beauty ; one probably never seen in nature and yet it is difficult to see why it should lay any just claim to the divine. It may be extra-human but not super-human.

There can be *no* conception of the divine.

Winckleman thought he recognized in the Greek gods certain of the nobler features of the animal creation, indicating that they invested their deities with those attributes of the animals which they most feared.

There is a coloring of probability in this, as we know it was a favorite idea of many heathen nations to enshrine their deities in the forms of animals.

Winckleman's idea of the Greek seems to be a refinement of the pagan, in the endowment of the countenance of man with the attributes of the animal. He instances the head of Jupiter, in which he sees the forehead of the lion.

But Sir Charles Bell gives what seems a more rational theory of the perfection of the Greek statue.

It was not found in the combination of individual beauties however excellent ; not in actual expression, since in many cases the gods were representented as superior to the emotions of the human heart and in the majesty of repose ; not in adopting from the animal anything that was not common to man ; but, in the exaggeration of those attributes which are purely human.

Hence, by observation we may discover how the excessive developement of certain features degrades and beautifies the human countenance, and *vice versa* how we may improve and ennoble it.

For example let us take those features or organs that are common to man and beast. Beginning with such teeth as are exposed to view ; exaggerate the incisors, and we have the teeth, appearance and expressive of the graminivorous animal.

Enlarge and make prominent the canine teeth and the seizing and tearing propensities of the carnivorous animal are developed; give undue breadth to the jaws and strength to the masticating apparatus and we have the face of a lion; increase the size and action of the levator muscles of the upper lip and we have the snarling and irascible expression of the tiger; give undue prominence to the lower part of the face; the intellectual character is degraded; the animal predominates.

Reverse now these suppositions, modifying and softening all these features to the minimum consistent with their nature and uses; add to these the features and develop such attributes of the man as he does not hold in common with the animal, and the face approaches the most exalted human idea of the divine.

Form the mouth therefore with a view to the intellectual exercise of speaking rather than for the gratification of the animal passion of feeding. Strengthen those facial muscles which are not found in the brute; whose sole object is human expression. Let the base be sustained by a well developed chin; crown the whole with the sign of intellect, a full forehead approaching to the perpendicular line of the face, and the human countenance becomes distinguished from the brute.

Some Physiognomists have carried this doctrine so far as to affirm that any resemblance to the brute in the human countenance implies degradation of character, and profess to discover in the man such characteristics of the animal as some particular feature or his whole countenance suggests. But this can hardly be true, for when we come to analyze the cause of those impressions we find that it is not, that any particular feature, or that the whole countenance, is, in form, strictly a likeness of the brute, but rather, that by the operation of the fancy alone, the animal is suggested.

It is this association of a human feature with the function of the brute which implies degradation.

This associating power of the mind constantly influences our opinions.

In the abundant caricatures of public men of the present day we see this verified. The skillful artist will take the most intellectual of faces and suggest the animal with but few strokes of his pencil, and without destroying the likeness. If a feature be exaggerated whose function be allied to intellect, we ennoble and dignify it; but the exaggeration of an organ whose function is purely animal degrades it, and it is incompatible with the superiority of the human countenance.

Of Beauty in the abstract, writers on art have been able to affirm only, that it is the reverse of deformity.

Sir Joshua Reynolds held, that every face had in it the elements of beauty, and that to attain that beauty, it was but necessary to soften some of the features, without destroying the likeness. While this may be true of a picture or a statue it is manifest that the doctrine cannot be applied to any extent to the living subject. Beauty in the human countenance is of two distinct characters;—beauty of form, and beauty of expression.

The former is cold, mathematical, uncompromising; the latter living, animated, divine. The former is predetermined and hereditary, governed by laws beyond our control; the latter is the fruit of the affections, the creation of ourselves.

The bones of the head determine its shape and in the normal adult admit of no modification. The muscles of the face govern the expression, and this beauty, an emanation of the soul, may be cultivated without limit.

As the soul is nobler than the body, so beauty of expression transcends that of mere shape.

As all our faculties develope with their exercise, so must the constant influence of the affections modify the expression and spiritualize every face.

Thus over the homeliest of features, judged merely by physical types, may be hung a veil of spiritual beauty which transfigures the physical, and compels all who behold the purified and irradiated face, to acknowledge in it a beauty not born of the dust, out of which the perishable body is made, but of that divine essence which constitutes the immortal Soul.

There is a doctrine advanced by phrenologists, that the configuration of the head undergoes a change under the protracted influence of the emotions, and the developement of the mental faculties; not alone that the expression of the face may be permanently altered, but that the cultivation of all the nobler faculties will work a transformation in the brain case; in the very bones themselves; while *per contra,* an abandonment to all the baser passions will increase its brutal character. These results will unquestionably be exhibited in the *expression* of the face, but its configuration as determined by the bones remains substantially true to its type through life.

If *any* modification takes place it is not sufficient to transform an ugly feature into one of beauty.

The frequent illustration of the aged couple living together in harmony until their habits, actions, voices, and looks correspond, proves nothing for feature, but all for expression.

As the bones of the face determine its type, and as beauty in the form is dependent upon their shape; observation teaches us that in a vast majority of the human species, this beauty in all the features is unattainable by the adult in his normal state.

In the growth of the face, as we have seen, the excessive developement of the jaws, alveolar processes and teeth, contributes more to the brutal aspect of the countenance than the exaggeration of any other feature.

Judged by any standard of physical beauty, great depth of face from the nose to the chin is a deformity. Protruding jaws and prominent teeth, with lips that find difficulty in covering them, is still worse; a receding lower jaw and chin is lacking in dignity, while a full lower jaw and retreating upper lip, takes from the mouth its intellectual character.

In a narrow jaw with two prominent front teeth, we see the rat-like look of the *rodentia.*

In the mouth and its immediate surroundings, caricaturists find the greatest scope for their pencils.

When Michael Angelo in his great picture of the Last Judgment, portrayed demons, he enlarged and exposed the canine teeth, and gave the savage and ferocious look of the tiger, an expression too, not at all at variance with human physiognomy.

These phases of man's countenance to a greater or less extent, are within the daily observatien of almost every individual.

When these things occur in the child, or when the loss of the teeth and alveolar processes supervene in the adult, the singular privilege is granted to the dentist, not only of redeeming that which is wanting, but in very many instances of attaining that which is artistically superior. The present advanced stage of Dental Science and Art, furnishes abundant means to measurably correct most of these deformities.

In the child of ten or a dozen years of age, the alveolar processes, and the consequent position of the teeth, may be moulded into almost any desirable form, and the whole contour of the lower part of the face changed.

The ability and good taste required of the dentist in practicing these higher branches of his art, must be founded on a knowledge of the anatomy of the muscles of the face, their functions, and the philosophy of their outward manifestations.

It is all-important in any attempt at restoration, that the muscles have free play.

Nothing is more palpably opposed to expression, than constraint. Hence it is often the case in our art that a decided improvement may be made in the form of a feature but its most pleasing expression is destroyed.

A naturally retreating upper lip may be filled out and its beauty greatly enhanced, when simply its shape is contemplated, but the graceful action of its muscles is interfered with and the nobler and more important beauty of expression is sacrificed.

Expression is then, of paramount consequence.

In this exhibition of the emotions upon the human countenance all the muscles connected with the *orbicularis oris*, are muscles of expression, and some of them seem to have no other office.

Sir Charles Bell, says, "The character of human expression in the mouth is given by the *triangularis oris* or *depressor anguli oris*, a muscle which I have not found in any of the lower animals. I believe it to be peculiar to man and I can assign no other use for it than that which belongs to expression"

"The *triangularis oris* arises from the base of the lower jaw and passes up to be inserted with the converging fibres of almost all the muscles of the side of the face, into the corner of the mouth ; it produces that arching of the lip so expressive of contempt, hatred, jealousy ; and in combination with the elevator of the under lip or *Superbus* and the *orbicularis*, it has a larger share than any other muscle in producing the infinite variety of motions in the mouth expressive of sentiment."

"In speaking there is much motion to the lower lip ; consequently much activity in the muscles which form the fulness of the chin, yet a remarkable variety is produced in the lines which mark the features about the upper lip, by the play of the different muscles which converge to the mouth from the margins of the orbits."

The levator muscles of the upper lip, produce at times the finest action ever witnessed about the mouth.

We are so familiar with these exhibitions that they pass unnoticed, yet if we were to try the experiment of reversing faces in our observation we should be struck with the wonderful activity of the human countenance.

None of these muscles are directly concerned in the expression of the meaner passions which govern the brute.

The office of the *Zygomatic* combined with the *Buccinator* is chiefly to raise and retract the angle of the mouth.

These muscles which are common to man, and the carnivora have much the same effect when in action. If therefore when the lip is raised by them, a long, prominent, and pointed canine tooth is exposed to view, a snarling expression is given, suggestive of the malignant passions.

Expression is not the result of the action of any single muscle, but rather of the combined action of the aggregate: and, not altogether by their contraction; but by the tension of some, and relaxation of others.

As the natural tones of the voice are universally understood without respect to speech, so do the movements of the features appeal directly to all; like the brand of Cain, the heart is indelibly stamped upon the countenance, and its emotions are read in a language which needs no interpreter.

(To be Continued.)

